# Effect of Different Pre-Treatment on the Microstructure and Intumescent Properties of Rice Husk Ash-Based Geopolymer Hybrid Coating

**DOI:** 10.3390/polym14112252

**Published:** 2022-05-31

**Authors:** Mohd Na’im Abdullah, Faizal Mustapha, Kamarul Arifin Ahmad, Mazli Mustapha, Tabrej Khan, Balbir Singh, Tamer A. Sebaey

**Affiliations:** 1 Department of Aerospace Engineering, Faculty of Engineering, Universiti Putra Malaysia, Serdang 43400, Malaysia; faizalms@upm.edu.my (F.M.); aekamarul@upm.edu.my (K.A.A.); balbir.s@manipal.edu (B.S.); 2 Department of Mechanical Engineering, Faculty of Engineering, Universiti Teknologi PETRONAS, Seri Iskandar 32610, Malaysia; mazli.mustapha@utp.edu.my; 3 Department of Engineering Management, College of Engineering, Prince Sultan University, Riyadh 11586, Saudi Arabia; tsebaey@psu.edu.sa; 4 Department of Aeronautical and Automobile Engineering, Manipal Institute of Technology, Manipal Academy of Higher Education, Manipal 576104, Karnataka, India; 5 Department of Mechanical Design and Production Engineering, Faculty of Engineering, Zagazig University, Zagazig 44519, Sharkia, Egypt

**Keywords:** rice husk ash, fire retardant, geopolymer, intumescent, coating

## Abstract

Despite the growing popularity of rice husk ash (RHA) in various applications, limited research has been devoted to identify the influence of silica content in RHA on the intumescent properties. The present work aims to introduce a novel and economical geopolymer hybrid fire retardant coating by utilizing the use of RHA. The silica from Rice husk (RH) was extracted using distilled water and hydrochloric acid as leaching agents and subjected to pyrolysis treatment. X-ray fluorescence (XRF) analysis indicated that RH that underwent HCl pre-treatment at 600 °C for one hour produced a high purity amorphous silica content of 93.92%. XRD measurements revealed that HCl pretreatment increased the crystallization temperature of RHA to 1000 °C and retained the amorphous state of silica for 2 h. In a fire resistance test, temperature at the equilibrium and time taken to reach 200 °C for sample S3 (93.92% wt. silica) showed 5.83% and 3.48% improvement compared to sample S1 (87.49% wt. silica). The microstructure analysis showed that sample S1 possessed bigger pores on the coating surface while an increment in silica content in sample S3 produced a dense foam structure. Results from a fire resistance test were supported by the Energy dispersive X-ray (EDX) analysis of the sample. The oxygen-to-carbon ratio of S1 and S3 coating samples were 1.695 and 1.622 respectively, which indicated that lower oxygen–to-carbon ratio in sample S3 coating resulted in better anti-oxidant properties. Interestingly, the increment of SiO_2_ content in RHA efficiently improved the compactness of the char layer, which resulted in a relatively higher fire-retardant efficiency. RHA proved to be a promising environmentally friendly strategy to replace halogenated fire retardant materials.

## 1. Introduction

The classic process of acid source, char former, blowing agent, and binder has been used in the vast majority of intumescent coatings in recent years [1]. It contains a large number of organic compounds, and the foam that develops during in-tumescence is mainly carbonaceous char. Silicon-based coatings containing expandable graphite and organoclay particles are among the more recent approaches. These systems have the advantage of producing a more mechanically and thermally stable residue after foaming, making them ideal to be applied at higher temperature areas or areas with higher concentrations of abrasive particles or corroding gases [2]. Fire-protective coatings for extreme conditions will continue to evolve as a result of increased demands, leading to the production of new materials. 

Geopolymers have emerged as one of the most exciting materials in recent years. Geopolymer binder (GB) is known for its outstanding thermal properties, so it has been used in many industrial applications and has drawn global marketing investments in many categories, such as resin, paint, binder, grout, cement, concrete, ceramic, panels and fiber-reinforced composites [3]. GB material has been verified to demonstrate outstanding fire resistance properties [4], high mechanical strength [5], and high durability [6]. As inorganic polymeric materials, geopolymers can be synthesized from various waste by-products. The aluminosilicate source in geopolymer materials are materials rich in alumina and silica content such as mine tailings [7], ashes [8,9], clays [10,11], or slag [12,13]. Some other natural and artificial silico-aluminates such as zeolite [14] and magnesium-contained minerals [15] have also been used as an important source of Si^4+^ and Al^4+^ ions in the geopolymer binding system. Normally, the total composition of Al_2_O_3_ and SiO_2_ is more than 70%, which is preferable in the reactive amorphous phase [16,17]. 

Aside from natural occurring minerals, rice husk ash (RHA), which is considered as an agricultural waste, has been proven as a good source of silica and has a vast potential in offering an alternative to commercial paint filler and as a raw material in the production of geopolymers [18]. Recent studies have reported that fire-resistant RHA-based geopolymer coatings managed to withstand flame exposure for an extensive period [19,20]. Research done by Basri et al. (2016), initiate that RHA-based geopolymer coating on mild steel plate to be in equilibrium state at 398 °C after 800 °C to 1000 °C heat been applied [21]. The geopolymer coating is able to reach thermal stability at 514.4 °C, which is reported as the lowest temperature in that study [21]. The researchers also claimed that the TGA curve results obtained by the selected RH-based geopolymer coating is similar with a metakaolin-based geopolymer in terms of thermal degradation and stability. Furthermore, the interaction between the ratio of RHA/alkaline activator (AA) and the concentration of sodium hydroxide (NaOH) plays an important role in affecting the fire resistance properties of RHA-based GB [21]. Since RHA mainly consists of silica, therefore the structure and property of RHA is also influenced by the nature of silica. RHA possesses a highly porous structure which would contribute to a material’s thermal resistivity [22,23]. Liang et al. (2022) revealed that the thermal conductivity of RHA-based geopolymer exhibits 0.1331 W/(m⋅K), which reflects good thermal insulation characteristics and better than the reported thermal insulation properties of other alkali-activated foamed materials systems at the same strength/density level [24].

The reactivity of RHA is based on its properties as an amorphous silica. The form of silica produced and the reactivity of RHA is dependent on several factors such as the incinerating temperature and time. Incineration temperature and time play a vital role in affecting the silica phase, whether it remains amorphous or is transformed into the crystalline form [25]. Furthermore, numerous studies have been carried out to investigate the effects of pre-treated rice husks (RH) on the RHA composition. The main objective is always to obtain a high silica content and it has been proven that by using acid as a pre-treatment process, the impure metallic contents in RH can be eliminated [26]. Several pre-treatments by using different types of acid applied to RH have been carried out. Hydrochloric acid (HCl), nitric acid (HNO₃), sulphuric acid (H_2_SO_4_), and citric acid are the acids commonly used to treat RH. The study by Bakar (2016) [27], proved that by using an HCL pre-treatment with 0.5 M at 60 °C for 30 min and constant stirring, the XRF testing resulted in 99.58% of SiO_2_. That HCl leached RHA has SiO_2_ contents higher than H_2_SO_4_, both leached and unleached. However, studies on the combined effect of temperature, exposure time and pretreatment on rice husk ash are rarely reported. Moreover, most of the previous literature discussed the effect of factors including the silica to alumina ratio on compressive strength of the geopolymer [3,8,13,14]. The majority of researchers were more interested in investigating the effect of factors on compressive strength as compared to other mechanical and thermal properties due to the fact that the most commonly used application of geopolymer is in building construction, especially concrete [5,6].

In addition, RHA-based geopolymers have a high potential to be resistant to thermal exposure [28], and this has led to the improved thermal stability and fire retardancy of the coating [4]. Geopolymers possess high thermal stability and fire resistance properties and would be an ideal alternative to conventional intumescent paint due to their unique properties. However, there is still not as yet well-established research about the combined effects of the pretreatment and pyrolysis process on the RHA-based geopolymer hybrid coating foaming process or its intumescent properties. The objective of this research is to study the relationship of different pre-treatments on the production of silica content from RHA while maintaining its reactive amorphous phase and its effects on the intumescent properties of the geopolymer hybrid alkyd coating. The properties studied were the chemical composition and mineral phase of RHA. The RHA samples with the highest and lowest silica content were then fabricated and subjected to a fire resistance test. The surface morphology of the hybrid coatings was then analysed and the best overall performance will be suggested as the coating for fire resistance application.

## 2. Materials and Methods

### 2.1. Sample Preparation

The RH in this research was obtained from a local rice factory, which is located at Tanjung Karang, Selangor, Malaysia. The species of the RH obtained was Oryza sative (Asian rice), which is vastly cultivated all over Asia. The RH was vetted to remove contaminations such as sands, rocks, and rice straws. In this process, the RH was washed and soaked in distilled water for 2 h in a large container to remove dirt and possible contaminations. Clean RH can be obtained as it floats on the surface of the water and was transferred to a sieve for the drying process and was left to dry for 24 h at room temperature. The chemical composition of untreated RHA is shown in Table 1. SiO_2_ was found to be the major constituent in RHA. Other components present included PdO, Al_2_O_3_, and Fe_2_O_3_.

**Figure 1:**
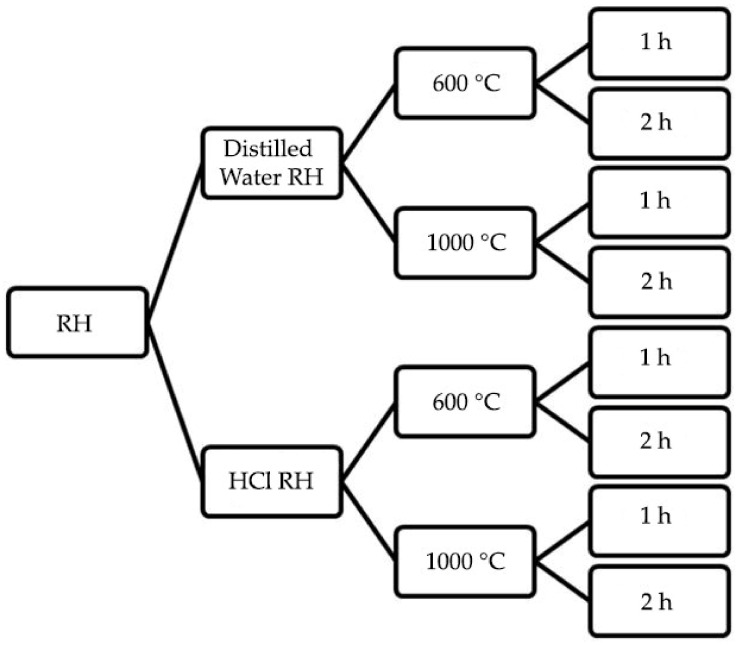
RH samples preparation process.

Two types of pre-treatments were used for rinsing the RH to determine the effect on RHA silica content. Incineration parameters were set based on temperature changes from black RHA to white RHA, which is highly preferable due to its low carbon content. A pure white RHA was produced at a range of 600 to 1000 °C [25,26,27]. According to Steven et al. (2021), the minimum incineration temperature to produce white RHA was 600 °C for 9 h [25]. Xu et al. (2016) revealed that a high purity amorphous silica from RHA was produced at incineration temperature of 1000 °C for 7 h [26]. As the main objective of this study is to produce an economical RHA-based geopolymer coating, the incineration parameters were set at the lowest incineration temperature to produce white RHA (600 °C) and by increasing the incineration temperature from 900 °C to 1000 °C while reducing the incineration period from 7 h to 1 h and 2 h. The main reason was to reduce the amount of energy used to extract the silica from RH. The process flow for RH samples of different pre-treatment through the chemical pre-treatment using distilled water and hydrochloric acid (HCl) and combustion period is illustrated in Figure 1.

For material characterization and microstructural analysis, RHA was categorized into eight samples subjected to different burning temperatures and periods, unleached and leached. The abbreviation for each sample is shown in Table 2.

### 2.2. Rice Husk Pre-Treated Using Distilled Water

For pre-treatment, distilled water was used to rinse the RH. Distillation removes all minerals from water, thus distilled water is inert and an ideal control element for the research project. Therefore, the sample rinsed with distilled water will be cleaner than using tap water, which can affect the outcome of the results (if there were minerals or live organisms in the water, this could lead to results that are biased and are not accurate). Distilled water was used in this experimental work during the rinsing of RH and HCl.

### 2.3. Rice Husk Acid Leaching Pre-Treatment

The second pre-treatment for RH is acid leaching using a 1M concentration of HCl. HCl that was used in this experimental work was purchased from Malay-Sino Chemical Industries (Perak, Malaysia). The optimum ratio that has been experimented with by other researchers revealed that 10 g of RH requires 100 mL, 1 M HCl solution [27]. A MR Hei-Standard (Heidolph, Illinois, USA) magnetic stirrer was used to heat the beaker. HCl solution was mixed with RH and stirred for thirty minutes once the temperature reached 60 °C. A thermometer was used to ensure the temperature was 60 °C throughout the process. Once the stirring process was completed, RHA was filtered using a standard stainless-steel mesh screen and washed with distilled water to remove any remaining HCl solution and solid residue. Then the RH was evenly distributed on an aluminum foil plate and dried in an open environment for 12 h. For the incineration process, a WiseTherm Digital Muffle Furnace (Daihan, Gangwon, Korea) was used for incinerating dried RH to RHA with a controlled temperature of 600 °C and 1000 °C for one and two hours, respectively.

### 2.4. Preparation of Geopolymer Hybrid Alkyd Coating

**Figure 2:**
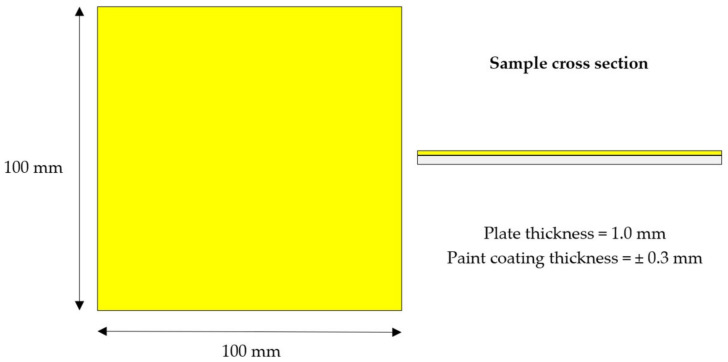
Geopolymer binder hybrid coating sample schematic diagram.

An optimum ratio of NaOH and NA_2_SiO_3_, which acts as the activated alkaline (AA) solution, was mixed with RHA. Na_2_SiO_3_ solution was purchased from LGC Scientific (Selangor, Malaysia). NaOH pellets with 97% purity were provided by Merck KGaA (Darmstadt, Germany). Concentrations, which are expressed as molarity, 8 M of NaOH solution, were prepared based on the number of pellets dissolved in de-ionized water. Na_2_SiO_3_ was added into the NaOH solution at a ratio of 5.5 to form an optimal AA solution [4] (the optimal ratio of AA to RHA is 2.5) to form an optimal GB. The quality of surface preparation is the key factor that affects paint performance. It is essential to remove all soluble salts, oil, grease, and other surface contaminants to assure the good and permanent adhesion of the paint system. The surface preparation for the fabricated sample is in accordance with ISO 8504-3:2018 Preparation of steel substrates before the application of paints and related products—Surface preparation methods—Part 3: Hand and power-tool cleaning (ISO, 2018). A yellow (BS2660-0001 Canary) super gloss finish 6000-S Alkyd-based paint (Kossan, Selangor, Malaysia) was used throughout this experiment and was coated on 100 mm × 100 mm × 1.0 mm mild steel. Three coats of paint were applied on the mild steel until a ± 0.3 mm coating thickness was attained. The hybrid geopolymer formulation was applied onto a steel plate according to ISO 4618:2014—paints and varnishes—Terms and definitions (ISO, 2014). A schematic diagram of the geopolymer binder hybrid coating is shown in Figure 2.

### 2.5. Material Characterization and Microstructural Analysis

Samples were analyzed using three important analyses, which were X-ray fluorescence (XRF) (Shimadzu, Kyoto, Japan), X-ray diffraction (XRD) (Philips, Malvern, United Kingdom), and scanning electron microscopy (SEM) (Hitachi, Tokyo, Japan). XRD is an analysis used for phase identification of the RHA sample. The dried RHA sample residues of approximately 5 mg to 10 mg were analyzed using a Philips PW3050/60, which records the intensity of the reflected X-rays rotating at each angle 2theta (2θ) range from 20° to 80°. PANalytical X’Pert HighScore software version 4.9 (Malvern Panalytical Ltd, Malvern, United Kingdom) was used to identify the phase, diffraction pattern analysis, and pattern treatment of the diffraction data obtained from the XRD instrument. This analysis is to ensure that the produced RHA from the different pre-treatment processes is still in amorphous form. The chemical components for each sample were then identified and measured in weight percent (wt. %) using XRF analysis. In this research, the main focusing element is silica, (SiO_2_) which is important for the thermal stability of coatings and fire-retardant performance.

**Figure 3:**
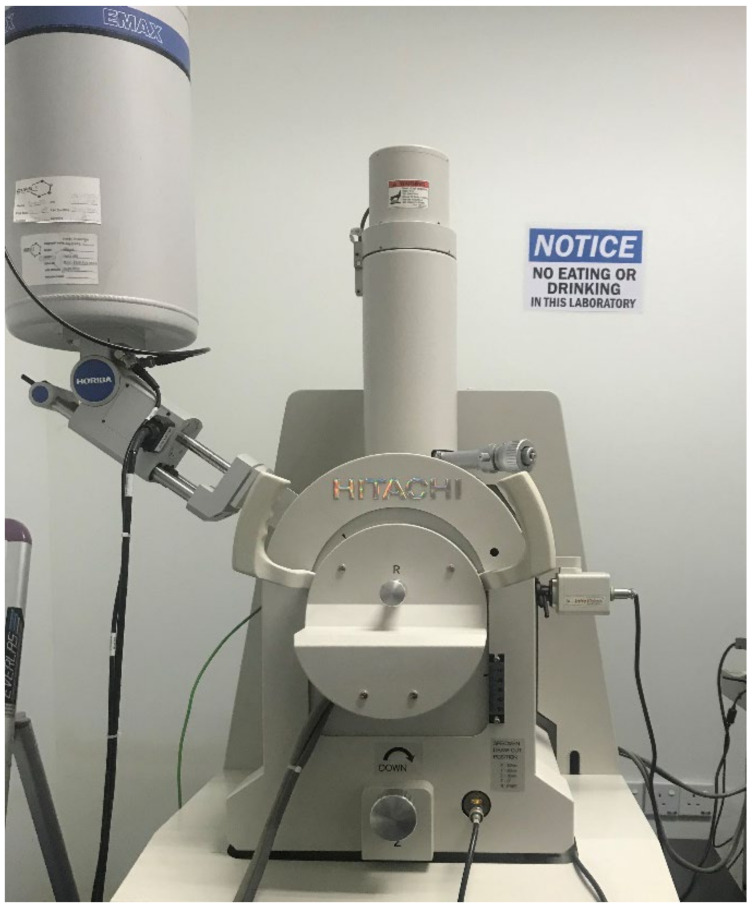
A scanning electron microscope (SEM).

SEM is used to analyze the structure of RHA and the char layer of coating samples after the fire resistance test. The microstructure was outlined by exposing the surface structure of the material underneath a microscope with at least 25× magnification. SEM imaging was undertaken using a Hitachi S-3400 N (Hitachi, Tokyo, Japan) for the particle study and compound representation of the samples as shown in Figure 3. It was conducted at 15 kV. The importance of the SEM result is used to prove and support the information about the phase or structure of RHA conducted in the fire resistance test.

### 2.6. Fire Resistance and Furnace Test

**Figure 4:**
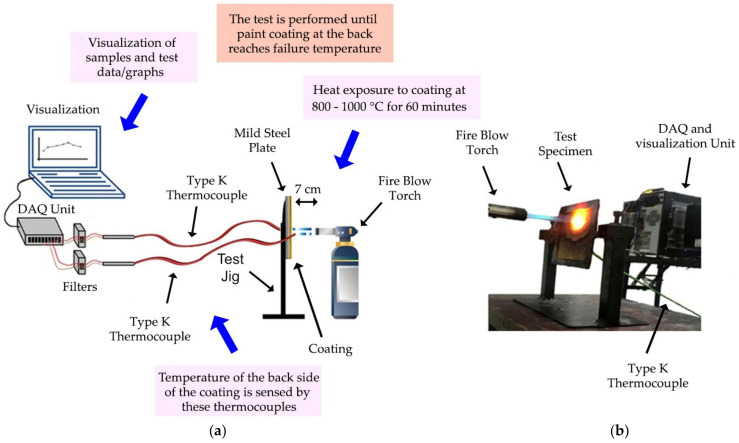
The fire resistance test. (**a**) Fire resistance test illustration; (**b**) Fire resistance test setup.

To evaluate the coating performance, samples were observed and characterized through two different fire tests. The fire resistance test was performed to evaluate the coating char thickness and the furnace test was performed to study the expansion rate of the char layer formation. The fire resistance test was performed until the backside of the paint coating reached failure temperature. The fire resistance testing was designated using a thermo-couple that acted as a temperature sensor to check the temperature at the backside of the coating samples. The test is carried out by using two Type-K thermocouples connected to Agilent 34970A data acquisition (DAQ) sensor (Agilent, Santa Clara, CA, United States). The thermocouples are attached at the backside of the steel plate coated with fire retardant coating (to measure the heat on the backside) and at the front of the surface of the coating (to measure the initial temperature produced by the fire blow-torch). The coatings are exposed to heat at the temperature of 800 to 1000 °C for 60 min, which complies with BS 9999: 2017 Code of practice for fire safety in the design, management, and use of buildings (BSI, 2017). The test produced time/temperature graphs and the visual effects for all samples. The illustration and actual setup of the fire resistance test is shown in Figure 4.

**Figure 5:**
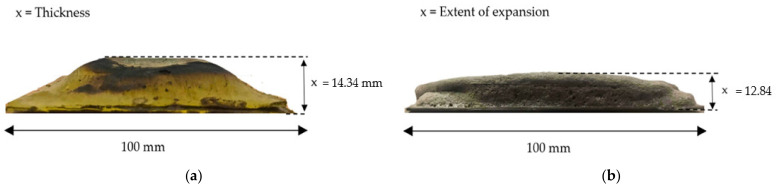
Char layer measurement. (**a**) Char layer thickness; (**b**) Char layer extent of expansion.

The measurement of the coating char thickness was obtained from the fire resistance test, while the expansion rate of the coating char layer was obtained by a furnace test, which was carried out to examine the expansion rate of the coating char layer. Three samples were tested for each testing to obtain the average value. These samples then underwent EDX analysis for the anti-oxidant properties of intumescent coating. The difference between the furnace test and fire resistance test is the heat direction exposed to the coating samples. The fire resistance test is done by using a blowtorch flame, as discussed in the previous paragraph. Heat is only applied to the front side of the coating samples, while in the furnace test, samples were heated uniformly from all directions, which increased the heat and mass transfer of the coating. The temperature was kept constant at 50 °C for 15 min and then was raised to 800 °C for 30 min of dwell time to expose heat to the samples completely. Subsequently, the sample was cooled for about 30 min to prevent the samples from cracking. The char coating thickness and the extent of expansion measurement for the coating sample is shown in Figure 5.

## 3. Results and Discussion

### 3.1. RHA X-ray Fluorescent Characterization

In this research, the main focusing element is silica (SiO_2_), which is important for thermal stability and fire-retardant performance. Table 3 shows the percentage elements for unleached and HCl leached RH with a control combustion of 600 °C. Based on the XRF result in Table 3, SiO_2_ content for both leached RH is higher than unleached RH. The highest concentration of silica (SiO_2_) content was found in sample S3 with 93.92 wt.% concentration of SiO_2_. HCl treated RH samples proved to be effective by removing some metals to a lower level. Among the metallic element, the percentage of potassium (K_2_O) is the highest in sample S2 with 0.82 wt.%. This result is consistent with the study by Xu et al., (2018) [26], who concluded that HCl is superior for removing metallic elements in RH compared to other acids. This is because the chloride ion (Cl^−^) from HCl protonates the silicon and forms silicon chloride (SiCl_4_) during the leaching process. SiCl_4_ is insoluble, therefore Si was not removed during the leaching process. Acid leaching treatment decreased the K_2_O element in RH due to the major agent responsible for the existence of unburnt carbon in ash. By eliminating K_2_O, the formation of black particles is avoided, hence resulting in a whiter RHA. In the unleached samples, the impurities increased from 0.80% to 0.82% as the incineration time increased. The thermal decomposition of K_2_O caused the surface of the RHA particles to begin to melt. The melted particles subsequently stopped the emissions of O_2_ and CO_2_, which increased the unburnt carbon composition content [29].

Table 4 shows the percentage elements for unleached and HCl leached RH with a control combustion of 1000 °C. Based on the XRF results, a higher content of SiO_2_ is found in both leached RH than the unleached RH. The highest concentration of SiO2 content was found in sample S8 with the SiO_2_ concentration of 92.10% wt. The drop in the level of K_2_O at 1000 °C silica lead to the formation of potassium polysilicate (K_2_SiO_3_) with residual carbon, hence the available K_2_O is reduced. The incineration time did not substantially increase the SiO_2_ composition for unleached samples. Instead, the percentage of SiO_2_ decreased as the incineration time increased from 1 h to 2 h. This result is supported by Xu et al., (2018) [26], who highlighted the transformation phenomenon of silica from an amorphous stage to crystalline related to the existence of the minor elements in RHA such as Na, K, Mg, and Ca, and thus leading to the decreased transformation silica temperature [26].

RH which underwent HCl pre-treatment at 600 °C for 1 h (Sample S3) produced the best amount of silica content in RHA (93.92%) and the least amount of carbon impurities. In conclusion, the incineration time of 1 h is sufficient to yield high SiO_2_ content in RHA. The increased incineration time is important in decreasing the other small amount elements such as Al_2_O_3_ and Fe_2_O_3_ but affected the silica content in RHA for unleached samples such as K_2_O in the unleached RHA. This circumstance caused the surface melting and accelerated the crystallization of amorphous silica to form cristobalite [30]. In comparison to the untreated RHA chemical composition in Table 1, results for treated RHA shown in Table 2 and 3 indicated that the silica content for all samples improved. 

### 3.2. RHA X-ray Diffraction Characterization

**Figure 6:**
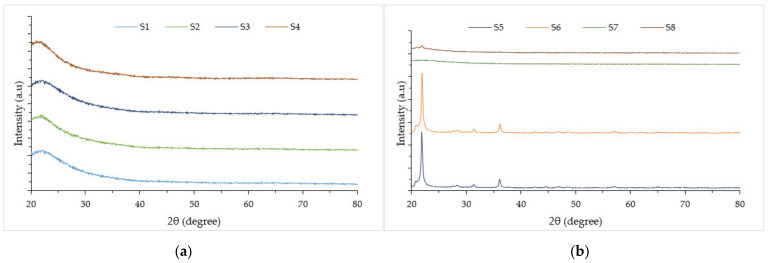
RHA XRD patterns at different incineration temperature. (**a**) Pre-treated RHA at 600 °C; (**b**) Pre-treated RHA at 1000 °C.

The RHA sample is categorized in the amorphous form subjected to the broad diffraction pattern with no diffuse peak. Figure 6 shows the XRD patterns of unleached and leached RHA at the incineration temperature of 600 °C.

The XRD for each sample revealed a similar pattern and broad diffused peaks with maximum intensity at a 2θ angle of 22°, indicating that the amorphous nature of the silica was observed. This peak position angle is mainly due to the occurrence of disordered cristobalite [31]. At 600 °C incineration temperature, unleached and leached samples were proved to be in amorphous form, which indicated that HCl treatment on RH did not modify the silica structure transformation from amorphous to crystalline.

Unleached RH for samples S5, S6 and leached RH for sample S8 showed the crystalline formed at the 1000 °C incineration temperature. This is due to the content of K_2_O in unleached RHA causing surface melting and accelerating the crystallization of amorphous silica to form cristobalite. Traces of cristobalite were found in sample S6 with the highest counts of 25,195.24 at the position of 21.8864°. White crystalline ash is undesirable for paint production due to its smaller surface area and is less reactive due to electrical neutrality. Meanwhile, for S8, a small peak on the 2θ angle of 22° was observed due to the impure form of silica, which lowered the temperature limit to transform into the crystalline form. Traces of Zeolite beta are found at the position of 21.9490. Zeolites are hydrated crystalline aluminosilicates with open three-dimensional (3D) framework structures, made up of SiO_4_ and AlO_4_ tetrahedrals linked by sharing their oxygen atoms to form regular intra-crystalline cavities and channels of atomic dimensions [32].

Meanwhile, sample S7 showed its condition still in the pure form of amorphous silica, which required a higher incineration rate to be transformed from amorphous into the crystalline form. As reported by Kwan and Wong (2020), leaching RH with acid solution reduced calcium, potassium, and sodium oxide impurities from the chemical composition of RH drastically. Consequently, not only the silica purity improves but also ash remained at the amorphous formed up to 1000 °C, whereas untreated RH transformed into crystalline formed at temperatures above 854 °C [33]. According to XRD measurements, it can be concluded that HCl pretreatment improved the crystallization temperature of RHA to 1000 °C and retained the amorphous state of silica for 2 h.

### 3.3. Coatings Fire Retardant Performance Comparison

Based on the XRF and XRD analysis on RHA, the optimum RHA obtained is in sample S3, which produced the highest amount of silica content in RHA, and the least amount of carbon impurities and amorphous silica with a purity of 93.92%. The thermal properties of the formulated GB are highly influenced by the SiO_2_ content of RHA and the form of silica. Amorphous form silica has a uniform pore structure, large specific surface area and higher porosity than other inorganic particles, which makes it possess good absorption and catalysis properties [34]. Even though sample S5 possessed the lowest silica content from XRF analysis, the XRD measurement revealed that the incineration period and temperature caused the phase of RHA to transform from amorphous to crystalline. Research by Zaffar et al. (2022) showed that a crystalline silica in RHA will lower the thermal resistance value [35]. The introduction of silica additive in paint helps to enhance the formation of a more compact and intumescent char layer and then effectively reduced the heat and smoke released, thereby enhancing the fire-retardant properties and smoke suppression properties of coatings [36,37].

**Figure 7:**
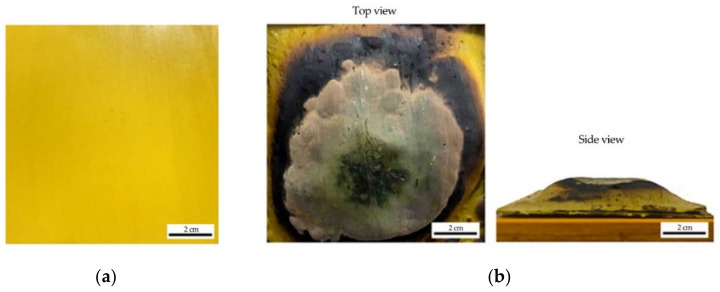
Sample S3 fire resistance test. (**a**) before; (**b**) after.

As the main objective of this research is to study the effect of silica content in RHA on the intumescent properties of the coating, sample S1 and S3 were chosen to be further investigated in fire resistance tests, as the results from XRF and XRD analysis show that these two samples obtained the highest and lowest silica content in RHA, while retaining its amorphous phase. The test was carried out by following the UL-1709 standards (UL 1709, 2017). The results were compared in terms of the optimum value of temperature at equilibrium (TAE) and the time taken to reach 200 °C (TT200). Both samples were coated with 0.3 mm GB hybrid coating. The ratio of GB to paint is obtained from the optimization model established by previous research by Abdullah et al. (2021). The optimum TAE and TT200 was achieved by using the composition ratio of GB = 48.625 and paint = 60.125 [4]. This formulation significantly contributed to a better fire protection performance due to the formation of intumescent. This circumstance helps to protect the mild steel substrate, thus affecting the temperature at equilibrium and the time taken to reach 200 °C. Figure 7 shows the fabricated sample before and after fire resistance test.

**Figure 8:**
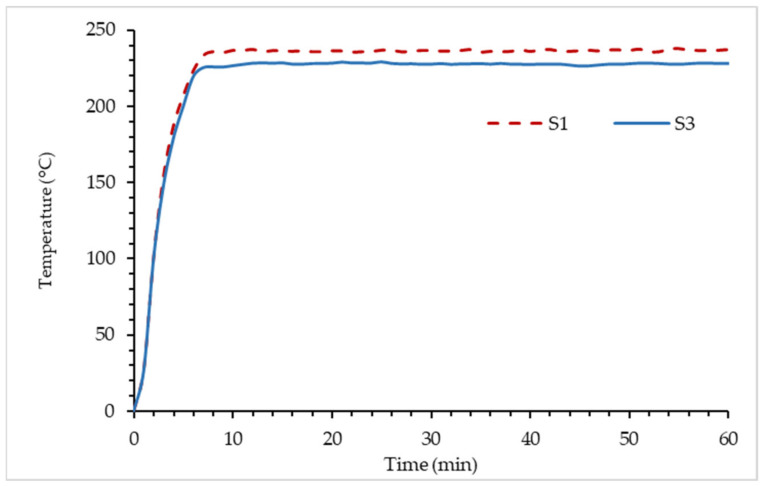
Graph temperature versus time in fire resistance test for samples.

Plotted graph of the temperature versus time for sample S1 and S3 is shown in Figure 8. From the graph, the result indicated that the silica in both samples S1 and S3 contributed to the formation of the char layer which affected the equilibrium temperature. The GB composition for both coatings resulted in a similar inclination for TT200 after eight minutes of the fire resistance test. This was due to the rate of char formation for both samples being influenced by the GB composition of the coatings. The effect of silica content can be observed at 223 °C as sample S3 is able to retain its TAE while sample S1 TAE continues to rise until 236 °C before it starts to retain its TAE. Mohd Basri et al. (2021) revealed that once exposed to fire, a lower silica content RHA-based geopolymer resulted in a weaker char strength due to the amount of water content evaporated, and generated pressure in the pores of the material and microvoids, which causes the coating to crack [38]. In the fire resistance test, it was observed that sample S1’s coating was unable to withstand the rapid temperature increase and experienced significant mass transfer out from the char, which resulted in higher TAE due to weaker char layer strength. Sample S3 showed 5.83% improvement in TAE compared to S1. While for TT200 analysis, sample S3 and S1 recorded 316 s and 305 s, respectively, to reach 200 °C. For TT200, sample S3 showed 3.48% improvement when compared to S1. It was also observed that the main difference between samples S1 and S3 were the char layer thickness and compactness. The result is in good agreement with the study by Beh et al. (2019), which showed that the char layer’s thickness influenced the coating’s fire safety efficiency [39]. The correlation between the char layer’s thickness and the equilibrium temperature will be further discussed in the coating characterization comparison. 

### 3.4. Coatings Characterization Comparison

In order to evaluate the coating performance, samples were being observed and characterized through two different fire tests. The fire resistance test was performed to evaluate the coating char thickness and the furnace test was performed to study the expansion rate of the char layer formation.

#### 3.4.1. Char Thickness of the Coating Char Layer

**Figure 9:**
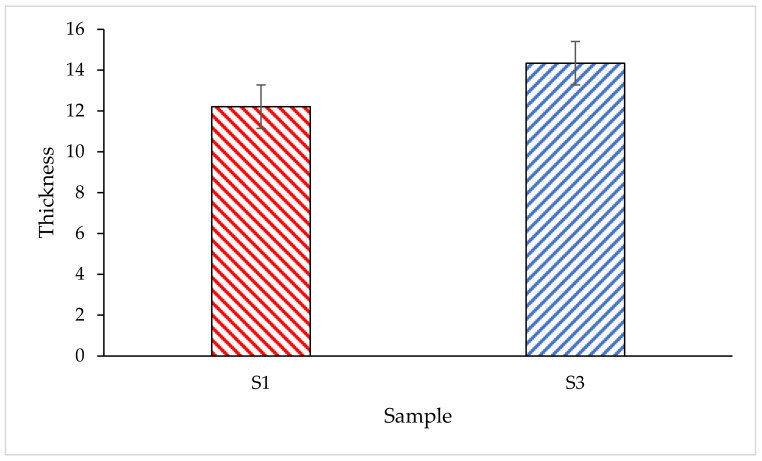
The thickness of char layer after fire resistance test.

The thickness of the char layer after the fire resistance test is shown in Figure 9. It was observed that the char formation thickness for S3 is 14.34 mm and it is 12.21 mm for S1. An improvement of 2.13 mm char formation thickness was observed with a difference of 3.61% in TT200 response and 1.83% in TAE response when S3 is compared to S1. The thickness of the intumescent char layer affected the fire protection performance of the coating, and a correlation between the thickness of the char layer and the equilibrium temperature was noted. This is in agreement with the research by Pimenta et al. (2016) [40], therefore it can be concluded that the volume of intumescence formed is the determining factor for thermal insulation. The coating’s performances decreased in the same order as the intumescence thickness decreased.

**Figure 10:**
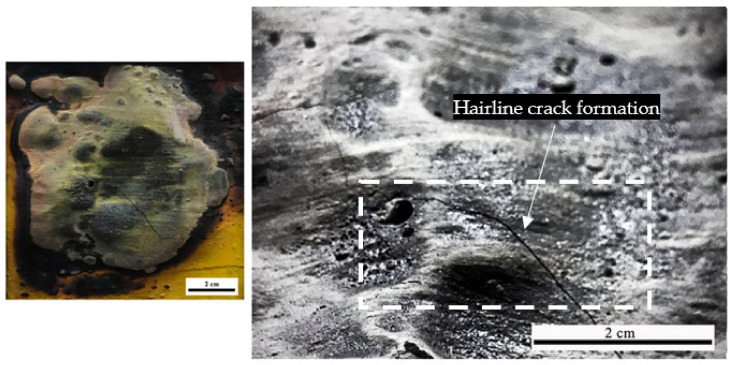
Sample S1 surface visual inspection.

Furthermore, the hairline crack formation was observed on the S1 coating sample surface, which prevents the coating from fully expanding for protecting the steel substrate, as shown in Figure 10. S1 coating was unable to withstand the rapid temperature increase in the fire resistance test and experienced significant mass transfer out from the char, which increased the heat, and transferred it into the metal surface. According to Wang et al. (2020) [41], the increased amount of SiO_2_ content produced a denser and a lesser pore residual char which enhanced the strength of the char layer once exposed to heat. Thus, this condition resulted in a more compact and harder char layer. A similar study by Mohd Basri et al. (2021) also revealed that lower silica content in RHA-based geopolymer leads to fewer pores for char layer expansion [38].

#### 3.4.2. Expansion Rate of the Coating Char Layer

**Figure 11:**
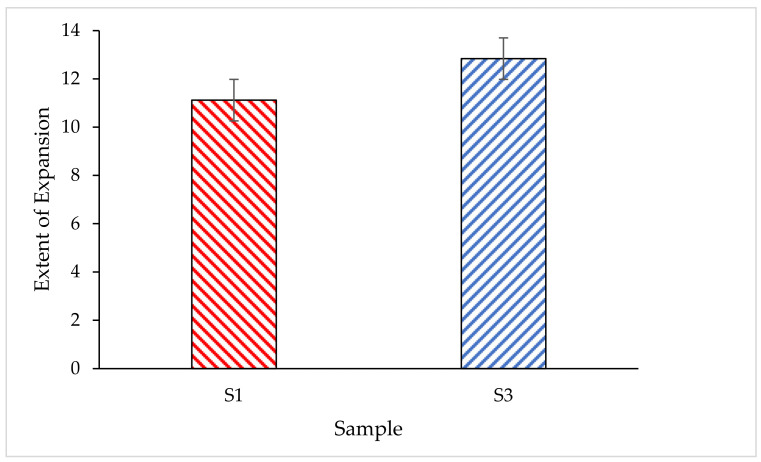
The extent of expansion of samples after the furnace test.

Figure 11 shows the graph of the extent of expansion of coating for both S1 and S3 coating samples. It was observed that the expansion of char for S1 was 11.12(×) and for S3 it was 12.84(×). The expansion of char observed was almost the same as the formulation for both coatings, which was similar to a 48.625% addition of RHA-based GB. Thus, it was found that the presence of RHA-based GB contributed to the effect of high swelling capacity which provided good protection to the substrate.

**Figure 12:**
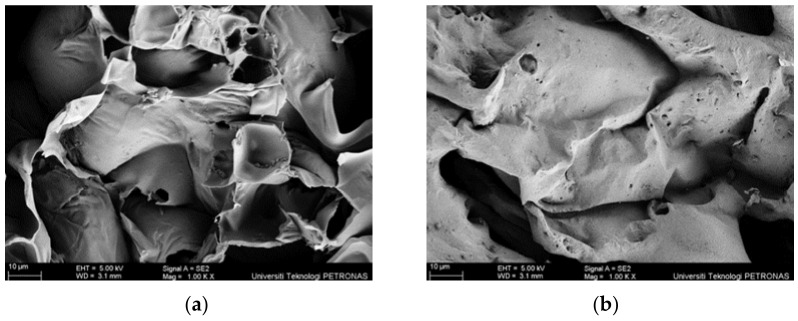
Surface SEM (**a**) S1 and (**b**) S3.

However, the fire protection performance of the intumescent coating did not only depend on the thickness of the char layer formed. The microstructure characteristic of the char layer formed also plays an important factor in minimizing heat penetration. According to Yew et al. (2014) [42], bigger pores on the coating surface encouraged a higher penetration of heat to reach the steel, which increased the temperature rise of the surface below the char layer. In addition, the pore sizes allowed oxygen to be diffused through the combustion reaction between the surface of the steel and the surface below the layer of char. The reaction between the heat and the char layer intensified and increased the char layer’s mass loss, which eventually caused the coating to fail and fall off from the substrate. The differences between these two char layers can be seen in Figure 12. As observed, the surface for sample S1 contained bigger pores and was less dense compared to S3. The dense foam structure on the sample S3 created a stable char layer, which led to the reduction of the heat from penetrating the steel plate substrate. This finding was supported by the result from the fire resistance, in which sample S3’s thermal performance improved by 3.61% for TAE and 1.83% for TT200 compared to sample S1.

**Figure 13:**
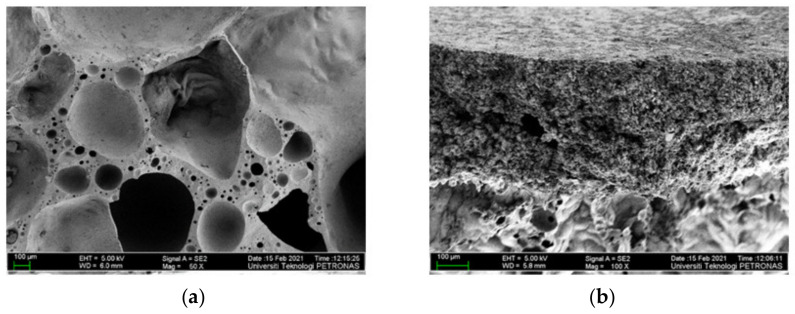
Cross-section SEM (**a**) S1 and (**b**) S3.

Furthermore, another major difference between sample S1 and S3’s coating samples was the char layer compactness. The char layer compactness was analyzed through SEM at the cross-sectional area, as shown in Figure 13. From observation, the char formed in sample S1 was fragile and contained big voids, which resulted in a relatively lower fire-retardant efficiency. The big voids resulted from the trapped gas evolved from the blowing agent. Meanwhile, for sample S3, it was observed that the char layer was more compact and smoother. It could be seen that SiO_2_ efficiently improved the strength and compactness of the char layer, as studied by Wang et al. (2020) [41]. This finding supported the result from the fire resistance of the previous section, which is the reason for a hairline crack formation that occurred on the S1 sample surface.

#### 3.4.3. Anti-Oxidant Properties of the Samples

The oxygen-to-carbon ratio is used to determine the anti-oxidant properties of intumescent coating [43]. Anti-oxidation of the coating is referred to as the ability of the coating to reduce the concentration of free radicals that required a flame reaction for self-sustaining. Better anti-oxidant properties are demonstrated by coatings that have lower oxygen-to-carbon ratios. That is because the resonance of the carbon bonds neutralizes more free radicals by donating an electron to stabilize them. The intumescent coating that has better anti-oxidant properties leads to better fire protection performance. It would be able to protect the underlying steel plate from the spread of fire and thereby increase the efficiency of fire protection.

**Figure 14:**
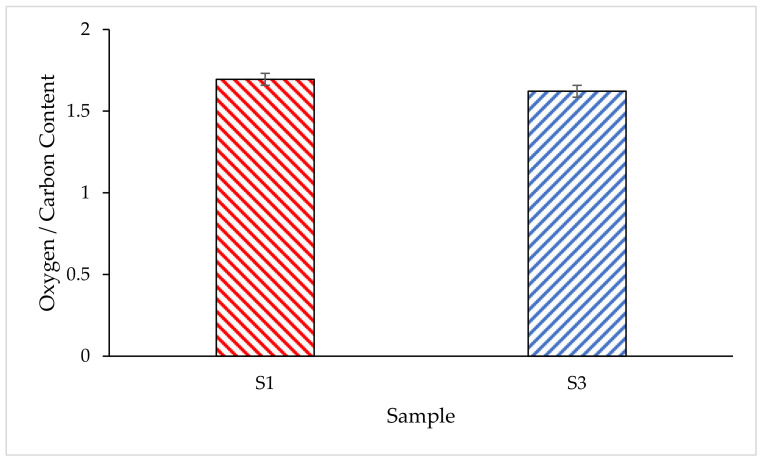
Oxygen/Carbon ratio of the char layer.

After the furnace test, the char layers formed by the coatings were sent for EDX analysis. Results obtained from the EDX analysis showed that sample S1 char contains 29.65% carbon (C) and 50.26% oxygen (O), while sample S3 contains 30.79% carbon (C) and 49.96% oxygen (O). Figure 14 shows the oxygen/carbon ratio in the char layers formed by S1 and S3 samples. Based on the result obtained in Figure 14, the oxygen-to-carbon ratio of S1 and S3 coating samples was 1.695 and 1.622, respectively. The result showed that the lower oxygen–to-carbon ratio of S3 coating was obtained over the S1 sample coating, which indicated that the S3 sample coating has better anti-oxidant properties. By referring to the result obtained in the fire resistance and furnace tests, the S3 sample coating showed better fire protection performance, and this matched with the result obtained from EDX analysis.

## 4. Conclusions

A different pre-treatment on RH was carried out to study the relationship of each pre-treatment on the chemical composition of the extracted silica content from RH. The intumescent properties of the RHA-based hybrid geopolymer coating was studied.

- It was observed that RH that underwent the HCl pre-treatment at 600 °C for 1 h (Sample S3) produced the best amount of silica content in RHA (93.92%) and the least amount of carbon impurities. An incineration time of 1 h was sufficient to yield high SiO_2_ content in RHA.
- From XRD analysis, at 600 °C incineration temperature, unleached and leached samples have shown the amorphous phase, while unleached RH for samples S5, S6 and leached RH for sample S8 showed the crystalline formed at 1000 °C incineration temperature. Raising the incineration temperature or extending the incineration duration can reduce the silica concentration and lead to accelerated formation of crystalline compounds.
- The comparison in thermal properties between RHA-based GB hybrid coating using optimum RHA and the lowest silica content RHA was conducted. Based on fire resistance results, TAE and TT200 responses for sample S3 showed 5.83% and 3.48% improvement compared to sample S1.
- SEM analysis revealed that the surface structure for sample S1 is more porous and less dense compared to sample S3. Furthermore, another major difference between S1 and S3 coating samples was the char layer compactness. Sample S1 was fragile and contained big voids while the char layer of sample S3 was more compact and smoother.
- It can be summarized that SiO_2_ efficiently improved the strength and compactness of the char layer, which resulted in a relatively higher fire-retardant efficiency. RHA has proven to be an effective alternative aluminosilicate source for geopolymer due to its intumescent formation and char layer properties.
- The RHA-based geopolymer hybrid coating has the potential to potentially improve building fire safety through passive fire protection and as thermal insulation material. Furthermore, utilizing silica content in RH is an innovative solution to transform “waste to wealth”.

## Figures and Tables

**Table 1:** Chemical composition of RHA.

Element	SiO_2_	PdO	Al_2_O_3_	Fe_2_O_3_	CaO	K_2_O	Cr_2_O_3_	MnO	LOI
(wt.%)	84.40	5.15	2.89	1.42	0.40	0.33	0.25	0.14	5.02

LOI = Loss on Ignition.

**Table 2:** Samples Categorization.

Time	Temperature 600 °C		Temperature 1000 °C	
	Unleached	Leached	Unleached	Leached
1 h	S1	S3	S5	S7
2 h	S2	S4	S6	S8

**Table 3:** Chemical composition of RHA samples at 600 °C.

Element	Sample			
	S1	S2	S3	S4
	(wt.%)			
SiO_2_	87.49	90.58	93.92	92.10
K_2_O	0.80	0.82	0.07	0.07
Al_2_O_3_	0.05	0.06	0.37	0.36
Fe_2_O_3_	0.04	0.03	0.03	0.03
CaO	0.37	0.43	0.15	0.12
TiO_2_	0.01	0.01	0.01	0.01
MgO	0.01	0.01	0.01	0.00
Na_2_O	0.01	0.01	0.01	0.00
MnO	0.01	0.01	0.01	0.00
LOI	11.21	8.04	5.42	7.31

LOI = Loss on Ignition.

**Table 4:** Chemical composition of RHA samples at 1000 °C.

Element	Sample			
	S5	S6	S7	S8
	(wt.%)			
SiO_2_	89.26	85.54	91.59	92.10
K_2_O	0.23	0.27	0.08	0.10
Al_2_O_3_	0.10	0.04	0.39	0.31
Fe_2_O_3_	0.04	0.04	0.03	0.02
CaO	0.25	0.23	0.08	0.08
TiO_2_	0.01	0.01	0.00	0.00
MgO	0.01	0.01	0.00	0.00
LOI	10.10	13.86	7.83	7.39

LOI = Loss on Ignition.

## Data Availability

All data are presented in the article.

## Abbreviations

AA	Alkaline Activator
Al_2_O_3_	Aluminum Oxide
ASTM	American Society for Testing and Materials
CaO	Calcium Oxide
DAQ	Data Acquisition
EDX	Energy Dispersive X-ray Spectroscopy
Fe_2_O_3_	Ferric Oxide
GB	Geopolymer Binder
HCL	Hydrochloric Acid
ISO	International Organization for Standardization
K_2_O	Potassium Oxide
LOI	Loss of Ignition
K	Potassium
MgO	Magnesium oxide
Na	Sodium
Na_2_SiO_3_	Sodium Silicate
NaOH	Sodium Hydroxide
OH	Hydroxide
RH	Rice Husk
RHA	Rice Husk Ash
SEM	Scanning Electron Microscopy
Si	Silica
TAE	Temperature at Equilibrium
TGA	Thermogravimetry Analysis
TiO_2_	Titanium Dioxide
TT200	Time Taken to Reach 200 °C
Wt.	Weight (g)
XRD	X-ray Diffraction
